# Novel Insights into the Downstream Pathways and Targets Controlled by Transcription Factors CREM in the Testis

**DOI:** 10.1371/journal.pone.0031798

**Published:** 2012-02-22

**Authors:** Rok Kosir, Peter Juvan, Martina Perse, Tomaz Budefeld, Gregor Majdic, Martina Fink, Paolo Sassone-Corsi, Damjana Rozman

**Affiliations:** 1 Center for Functional Genomics and Bio-Chips, Institute of Biochemistry, Faculty of Medicine, University of Ljubljana, Ljubljana, Slovenia; 2 Medical Experimental Centre, Institute of Pathology, Faculty of Medicine, University of Ljubljana, Ljubljana, Slovenia; 3 Center for Animal Genomics, Veterinary Faculty, University of Ljubljana, Ljubljana, Slovenia; 4 Department of Haematology, University Medical Center Ljubljana, Ljubljana, Slovenia; 5 Institute of Physiology, Faculty of Medicine, University of Maribor, Maribor, Slovenia; 6 Department of Pharmacology, University of California Irvine, Irvine, California, United States of America; 7 Diagenomi Ltd, Ljubljana, Slovenia; Institute of Genetics and Molecular and Cellular Biology, France

## Abstract

The essential role of the *Crem* gene in normal sperm development is widely accepted and is confirmed by azoospermia in male mice lacking the *Crem* gene. The exact number of genes affected by *Crem* absence is not known, however a large difference has been observed recently between the estimated number of differentially expressed genes found in *Crem* knock-out (KO) mice compared to the number of gene loci bound by CREM. We therefore re-examined global gene expression in male mice lacking the *Crem* gene using whole genome transcriptome analysis with Affymetrix microarrays and compared the lists of differentially expressed genes from *Crem*−/− mice to a dataset of genes where binding of CREM was determined by Chip-seq. We determined the global effect of CREM on spermatogenesis as well as distinguished between primary and secondary effects of the CREM absence. We demonstrated that the absence of *Crem* deregulates over 4700 genes in KO testis. Among them are 101 genes associated with spermatogenesis 41 of which are bound by CREM and are deregulated in *Crem* KO testis. Absence of several of these genes in mouse models has proven their importance for normal spermatogenesis and male fertility. Our study showed that the absence of *Crem* plays a more important role on different aspects of spermatogenesis as estimated previously, with its impact ranging from apoptosis induction to deregulation of major circadian clock genes, steroidogenesis and the cell-cell junction dynamics. Several new genes important for normal spermatogenesis and fertility are down-regulated in KO testis and are therefore possible novel targets of CREM.

## Introduction

In the last decade the high throughput techniques such as transcriptomics and proteomics have partially revealed molecular mechanisms and pathways that regulate normal male sperm development [1]–[6]. The use of genetically modified mouse models has brought some insight into the role of specific genes and the consequences of their deficiency [7]. To our knowledge Beissbarth *et al* [8] is the only study to date that applied transcriptomics to evaluate the global effect of *Crem* deficiency on spermatogenesis in mice. Affymetrix microarrays and suppression substractive hybridization were used in this study, which reported 126 and 158 differentially expressed (DE) genes between wild-type (WT) and knock-out (KO) testis of adult mice, respectively. Recently, Martianov et al. used chromatin immunoprecipitation coupled to next generation sequencing (ChiP-seq), and found that the CREM protein binds to over 5000 genomic loci in the mouse testis [9].

Spermatogenesis is a highly specialized process devoted to the production of mature spermatozoa [10]. It is regulated by stage specific gene expression programs that are carried out by specific transcriptional factors. Some of the most important changes occur at the stage of round spermatids, when the general transcriptional machinery is most active [11], [12]. During this time the CREMτ transcriptional activator protein is expressed at the highest levels [13], [14]. The importance of the *Crem* gene for spermatogenesis was confirmed through its inactivation. Homozygous *Crem* KO mice exhibit complete arrest of spermatogenesis at the stage of round spermatids and a several fold increase in the number of apoptotic germ cells [15], [16]. The *Crem* KO mice were the first animal model to mimic the round spermatid maturation arrest in humans [17], [18].

CREM [19] together with CREB (cAMP responsive element binding protein) [20] and ATF-1 (activating transcript factor 1) [21] belongs to the CREB family of transcriptional factors, that respond to cyclic AMP (cAMP) signaling and bind to cAMP responsive element (CRE) sites in promoters of selected genes. In contrast to ATF-1 and CREB that produce only activator isoforms, CREM can produce isoforms that have either activating or repressing functions, depending on the transcription of specific exons (Figure S1) [22], [23]. During male germ cell development the expression of CREM isoforms switches from repressors in pre-meiotic cells to an activating form (CREMτ) in post-meiotic cells, based on alternative splicing, alternative polyadenilation [24] and alternative translation initiation [13], [25], [26]. The switch occurs in pachythene spermatocytes when CREMτ mRNA is initially detected and begins to accumulate [19], [27]. The CREMτ protein starts to accumulate later, only in round spermatids at stages VII–VIII, just before overall transcription ceases in round spermatids at stage IX [28]. During this time CREMτ participates in up-regulation of haploid genes which is important for structural changes that occur throughout spermiogenesis and several of these genes have been shown to have CRE or CRE like elements [29]–[31]. Among these are also genes from metabolic pathways, such as *Cyp51* from cholesterol biosynthesis [32]–[34].

Due to our continuous interest to understand the physiological roles of *Crem* isoforms and their effect on downstream pathways [34]–[37] we re-examined the transcriptome of the *Crem* knockout mouse testis. Applying the Affymetrix GeneChip Mouse Gene 1.0 ST oligonucleotide microarrays we performed global transcriptome analysis and compared DE genes from testes of wild type and *Crem* KO mice to (a) genes where binding of CREM was determined by Chip-seq [9] and (b) lists of transcription factors available at TFCat transcription factor database [38]. This provided novel insights into the role of CREM family of transcription factors in spermatogenesis and enabled to distinguish between primary (direct) and secondary (indirect) effects of the CREM absence.

## Results

### Microarray and correlation analyses

**Figure 1:**
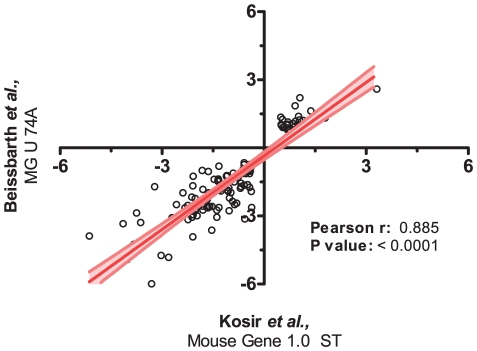
Correlation analysis. A Pearson correlation analysis was performed on fold-change values of 123 differentially expressed genes that were retrieved after comparing ours and Beissbarth *et al* datasets [8]. A correlation coefficient of r = 0.885 shows a high correlation between both datasets.

GeneChip Mouse Gene 1.0 ST microarrays (Affymetrix) were used to hybridize whole testis RNA from 5 wild-type (WT) and 5 *Crem* knock-out (KO) mice. Differential expression of genes was assessed by the LIMMA package at the false discovery rate of α = 0.05. Altogether, 4706 DE genes were discovered of which 1822 were down- and 2884 up-regulated in the KO testis (Table S1). Expression of a subset of DE genes was measured by qPCR to see whether fold-change increases as well as their direction correlate with microarray data. Table 1 shows genes measured by qPCR and comparison of log 2 fold changes between microarray and qPCR data. Multidimensional scaling of normalized data exposed clear separation between WT and KO animals (Figure S2). Differential expression of genes was also re-assessed from normalized data of 3 KO and 3 WT animals from Beissbarth *et al* (http://ibios.dkfz.de/tbi_old/crem/) [8], using the same analysis methods and conditions as for our data (Table S2). From this data, 131 genes were found to be down- and 46 up-regulated which represent only a small fraction of DE genes from our dataset. Comparison of both datasets showed that 123 (71%) (Table S3) of DE genes from Beissbarth et al. were also differentially expressed in our dataset and that their expression was highly correlated (Pearson correlation analysis coefficient of r = 0.885, see Figure 1).

**Table 1:** qPCR data validation.

		qPCR		Affymetrix	
Gene Symbol	Gene ID	Log 2 Fold-Change	p-value	Log 2 Fold-Change	p-value
*Arnt*	11863	1,58	0,0013	0,42	0,0000
*Atoh8*	71093	−0,17	0,2600	−0,69	0,0000
*Atf7ip*	54343	2,37	0,0100	0,4	0,0030
*Bach2*	12014	4,65	0,0300	0,78	0,0000
*Cbfa2t3*	12398	1,45	0,0004	0,32	0,0010
*Ctnnb1*	12387	1,34	0,0005	0,31	0,0010
*Dlx6*	13396	−4,64	0,0000	−1,16	0,0000
*Eid2*	386655	0,85	0,0030	0,41	0,0050
*Hnrnpab*	15384	2,23	0,0003	0,6	0,0000
*Mllt11*	56772	−0,27	0,1200	−0,77	0,0000
*Mllt3*	70122	−1,25	0,0000	−0,95	0,0000
*Ncoa3*	17979	1,50	0,0002	0,32	0,0000
*Nfe2l1*	18023	1,44	0,0004	0,22	0,0050
*Sin3a*	20466	2,48	0,0000	0,37	0,0020
*Smarcc1*	20588	1,33	0,0005	0,48	0,0010
*Suv39h2*	64707	−0,97	0,0030	−1,51	0,0000
*Tbpl1*	237336	2,26	0,0002	0,37	0,0030
*Tcf7l2*	21416	3,72	0,0000	0,68	0,0000
*Wwtr1*	97064	2,58	0,0001	0,82	0,0000
*Zscan21*	22697	2,35	0,0007	0,39	0,0000
*Nr4a1*	15370	−2,56	0,0000	−1,61	0,0000
*Pax5*	18507	−5,06	0,0000	−0,92	0,0000
*Paxip1*	55982	2,08	0,0005	0,36	0,0000
*Ring1*	19763	1,44	0,0000	0,29	0,0010
*Sall3*	20689	−0,23	0,3200	−0,31	0,0020
*Scrt1*	170729	−2,74	0,0000	−0,51	0,0000
*Catsper1*	225865	−5,92	0,0000	−4,38	0,0000
*Catsper3*	76856	−5,39	0,0000	−5,44	0,0000
*Akap4*	11643	−5,25	0,0000	−5,49	0,0000
*Kcnu1*	16532	−3,52	0,0000	−3,7	0,0000
*Camk4*	12326	−3,38	0,0000	−3,92	0,0000
*Smcp*	17235	−3,04	0,0000	−3,16	0,0000
*Tekt5*	70426	−0,49	0,0676	−3,43	0,0000
*Adcy10*	271639	−1,02	0,0002	−1,6	0,0000
*Gapdhs*	14447	−5,71	0,0000	−5,26	0,0000
*Spem1*	74288	−6,28	0,0000	−5,2	0,0000
*Prm2*	19119	−5,09	0,0000	−4,22	0,0000
*Prm3*	19120	−3,36	0,0681	−4,74	0,0000
*Tnp2*	21959	−6,23	0,0000	−5,46	0,0000

Several genes involved in different processes were measured by qPCR in order to determine expression levels and to validate the data gathered by DNA microarrays.

Since down-regulated genes could be a consequence of missing stages past round spermatids in *Crem* KO males [15], [16] the expression of a selected group of genes was measured also in pre-pubertal mice that were sacrificed prior to the development of elongated spermatids (day 30). Therefore both WT and KO mice had about the same set of germ cells present. qPCR shows that all measured genes were down-regulated also in pre-pubertal mice, confirming that CREM (and not the absence of cells) is responsible for the observed down-regulation (Supplementary Figure S3).

### Data comparison

**Figure 2:**
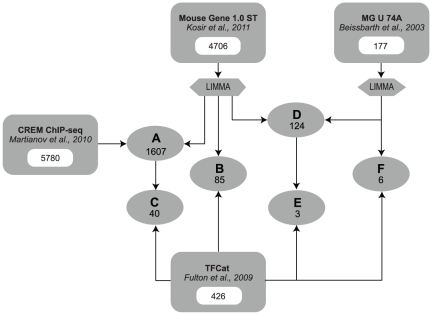
Data comparison. Differentially expressed (DE) genes were compared to the Crem ChIP-seq dataset from Martianov *et al* [9] from 2010 (dataset of genes that are bound by *Crem* in testis), the Affymetrix MG U 74A dataset from Beissbarth *et al* [8] from 2003 (dataset of DE genes between WT and KO mice testis) and the TFCat dataset from Fulton *et al* [38] (a hand curated database of transcriptional factors). **A** – DE genes that are bound by *Crem*; **B** – DE genes that are transcriptional factors; **C** – DE genes that are transcriptional factors bound by *Crem*; **D**, **E** and **F** are genes common to both analysis using Affymetrix microarrays. Numbers represent the number of genes in each dataset or the number of common genes between comparisons.

**Figure 3:**
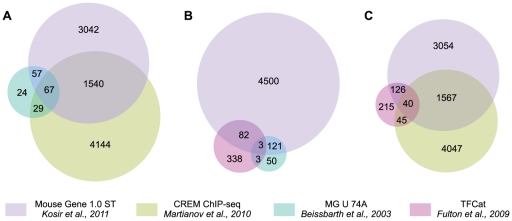
Euler diagrams representing genes from different datasets. Euler diagrams were drawn to visualize the comparisons of genes from different datasets. The size of the circles corresponds to the number of genes present in each dataset. Three comparisons were made in order to retrieve the data for further functional analysis of DE genes.

DE genes from our dataset were compared to TFCat transcription factor database [38] and to the CREM ChIP-seq database [9] using Venn and Euler diagrams. The numbers of genes present in each comparison as well as the number of all genes in each dataset are shown in Figure 2. Comparisons (A, B and C, Figure 2) enabled to determine which of the differentially expressed genes from our dataset are: (A) bound by the CREM protein (comparison A, Figure 2), (B) are transcriptional factors (comparison B, Figure 2) or (C) are transcriptional factors bound by CREM (comparison C, Figure 2). Comparisons D, E and F were used to evaluate the overlap between our dataset and the dataset from Beissbarth *et al* [8]. A more detailed overview of all comparisons between the four datasets is presented in Euler diagrams in Figure 3. We show that the absence of *Crem* deregulates a large number of genes in KO testis, however not all are direct targets of the CREM protein. Only 1607 (34%) fall into the cross section between our and ChIP-seq data (Figure 3, Table S4), while the rest (3099) are probably affected due to secondary effects of CREM absence. A comparison with the TFCat database [38] (Figure 3B and 3C) showed that 85 DE genes are transcription factors and are according to the analysis of GO terms (and KEGG pathways) involved in cell differentiation, development and spermatogenesis. Forty of these transcription factors are also bound by CREM (Figure 3C, Table S5). These transcription factors can explain the secondary effects of the CREM absence.

Based on the ChIP-seq data, we investigated the proportion of CRE, CRE-mismatch, half-CRE or no-CRE sites in promotes of genes that are bound by CREM. Surprisingly, only 180 (11.2%) had a full-CRE sequence, 496 (30.8%) a CRE element with one mismatch, 653 (40.6%) a half-CRE element while 278 (17.3%) had no-CRE element in their promoters. The same analysis was also performed on 4173 genes from ChIP-seq data that did not expose differential expression in our dataset (Figure 3, A). Interestingly, a similar distribution was obtained: 489 genes (11.7%) had a full-CRE sequence, 1104 (26.3%) had a CRE element with one mismatch, 1860 (44.6%) had a half-CRE element while 720 (17,3%) had no-CRE element in their promoters.

### Functional analysis of differentially expressed genes

**Figure 4:**
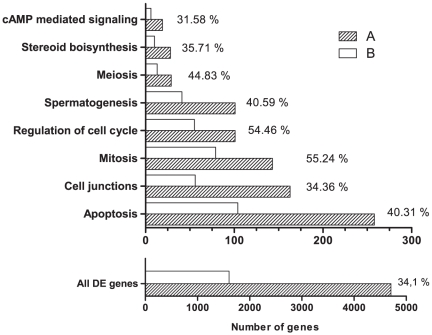
Functional characterization of genes deregulated in *Crem* KO mice. Biological processes deregulated in *Crem* KO mice together with the total number of genes deregulated in each process (A) and the number and relative level of DE genes that are bound by CREM (B).

The essential role of *Crem* in spermatogenesis is confirmed not only by complete absence of mature sperm cells in male homozygous KO mice [15], [16] but also by the large amount of DE genes found in KO animals. As noted above, not all differentially expressed genes are under direct control of CREM but are probably deregulated due to secondary effects of CREM absence. To functionally characterize the role of CREM, GO terms and KEGG pathways were tested for their enrichment. Figure 4 shows biological processes deregulated in *Crem* KO mice together with the number of DE genes, and the number and relative level of DE genes that are bound by CREM. Surprisingly, from the 1607 genes which were deregulated and were supposed to be bound by CREM, only 43% were down regulated. Since CREMτ is known to be a potent transcriptional activator during germ cells development [19], [39] one would expect that the majority of genes regulated would be down- and not up-regulated.

A positive control of our experiment and computational analysis was the deregulation of 101 genes involved in spermatogenesis, of which 41 are bound by CREM. Among them are several genes (22) whose absence in mouse models has proven their importance for normal spermatogenesis and male fertility. Importantly, they are all down regulated in *Crem* KO testis. The list of these genes, their function, fold-change and presence in other databases is shown in Table 2. Only 3 (15%) out of these 22 genes were also presented by Beissbarth *et al*, indicating that many genes important for normal spermatogenesis could have been missed in their study.

**Table 2:** Differentially expressed genes essential for spermatogenesis, epididymal maturation and fertilization.

Gene name	Gene symbol	Gene ID	Log 2 Fold change	Beissbarth*et al*	Bound by *Crem*	Fertility status*
Sperm maturation 1	*Spem 1*	74288	−5.20	No	No	Male infertility
Protamine 1	*Prm1*	19118	−5.53	No	Yes	Chimera male infertility
Protamine 2	*Prm2*	19119	−4.42	No	Yes	Chimera male infertility
Protamine 3	*Prm3*	19120	−4.47	Yes	Yes	Chimera male infertility
Transition protein 1	*Tnp1*	21958	−4.86	No	Yes	Male sub fertility
Transition protein 2	*Tnp2*	21959	−5.46	No	Yes	Male sub fertility
Testis specific serine kinase 6	*Tssk6*	83984	−3.53	No	Yes	Male infertility
Poliovirus receptor related 2	*Pvrl2*	19294	−0.41	No	Yes	Male infertility
Tektin-5	*Tekt5*	70426	−3.43	No	Yes	Male infertility
Adenylate cyclase 10	*Adcy10*	271639	−1.60	No	No	Male infertility
Glyceraldehyde-3-phosphate dehydrogenase, sperm	*Gapds*	14447	−5.26	No	No	Male infertility
Phosphoglycerate kinase 2	*Pgk2*	18663	−1.64	Yes	No	Male infertility
Cation channel sperm associated 1	*Catsper1*	225865	−4.38	No	No	Male infertility
Cation channel sperm associated 3	*Catsper3*	76856	−5.44	No	No	Male infertility
Cation channel sperm associated 4	*Catsper4*	329954	−2.07	No	No	Male infertility
Izumo sperm-egg fusion 1	*Izumo1*	73456	−1.75	No	Yes	Male infertility
A kinase (PRKA) anchor protein 4	*Akap4*	11643	−5.49	No	No	Male infertility
Proprotein convertase subtilisin/kexin type 4	*Pcsk4*	18551	−0.29	No	No	Male sub fertility
Potassium channel, subfamily U, member 1	*Kcnu1*	16532	−3.70	No	No	Male infertility
angiotensin I converting enzyme	*Ace*	11421	−2.42	No	Yes	Male sub fertility
calcium/calmodulin-dependent protein kinase IV	*Camk4*	12326	−3.92	No	No	Male infertility
sperm mitochondria-associated cysteine-rich protein	*Smcp*	17235	−3.16	Yes	No	Male infertility

* Fertility status was retrieved based on the information from [52].

**Figure 5:**
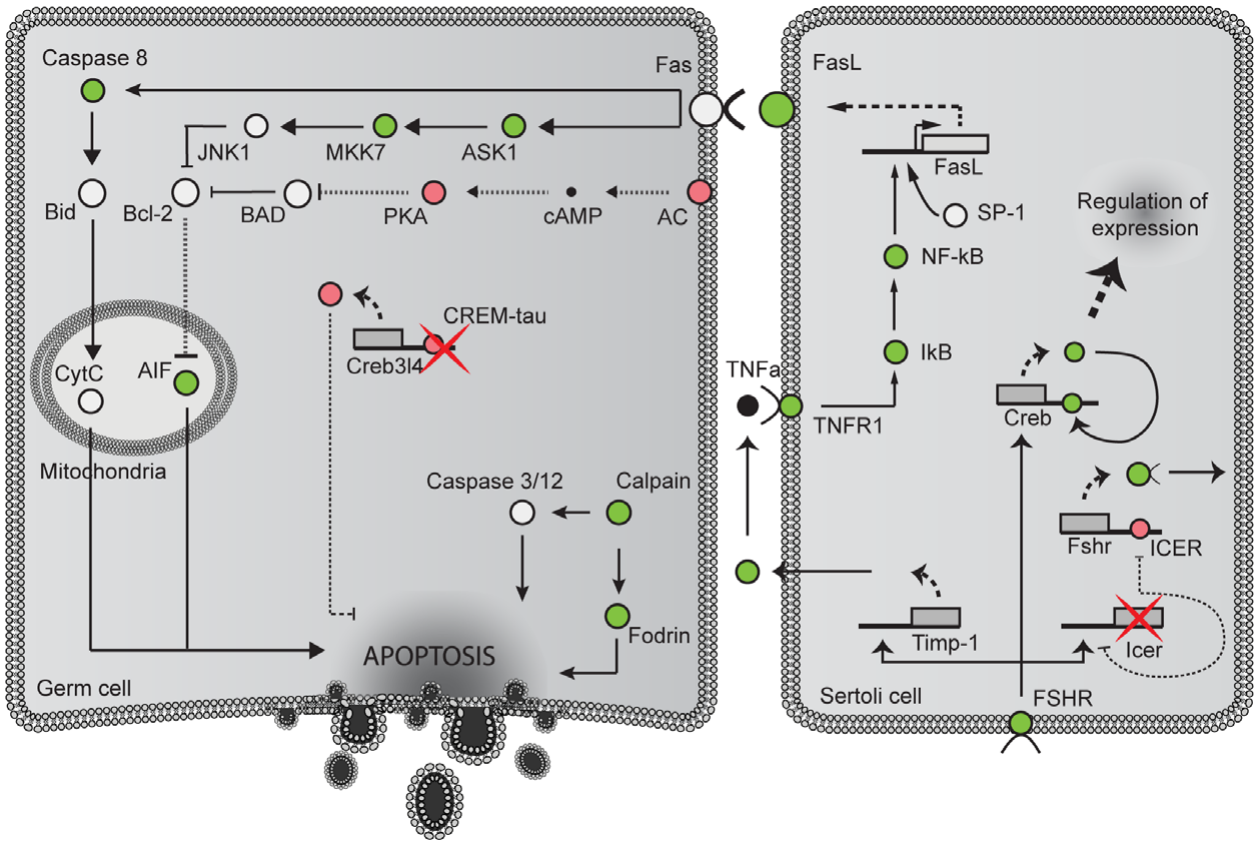
Apoptosis induction pathways in round spermatids. Synthesis of TNFα by germ cells induces the expression of Fas ligand on their surface which in turn activates the Fas receptor and caspase 8 or Ask1 mediated apoptosis in germ cells. The production of TNFα is mediated by the up-regulation of metalloproteinase's such as *Timp1*. Similar to Ask1, BAD also represses the action of the anti-apoptotic protein Bcl-2. BAD's activity is inhibited by phosphorylation with PKA which is down regulated in KO testis. Other apoptosis induction pathways through calpain and fodrin are also possible.

Another important feature of the *Crem* KO testis is the 10-fold increase in germ cell apoptosis [15]. In line with this fact, 258 genes, 104 of which are bound by CREM and are involved in apoptosis, are differentially expressed in our dataset. Both GO term and KEGG pathway analysis show up-regulation of apoptosis and the p53 signaling pathway, respectively. The activation of the Fas mediated apoptosis through Caspase 8 or *Ask1* pathways in germ cells could be a result of the up-regulation of *FasL* expression in Sertoli cells. Several other apoptosis mediating factors such as *Aif*, *Capn* (Capain) and *Spna* (Spectrin) were also deregulated in KO testis (Figure 5). The upregulation of *Fshr* and *Icer* could be partially explained by the absence of ICER protein, which is known to affect the expression of both of these genes [40], [41]. *Fshr* is important also for expression of *Timp-1*, which contributes to up-regulation of TNF-α.

**Figure 6:**
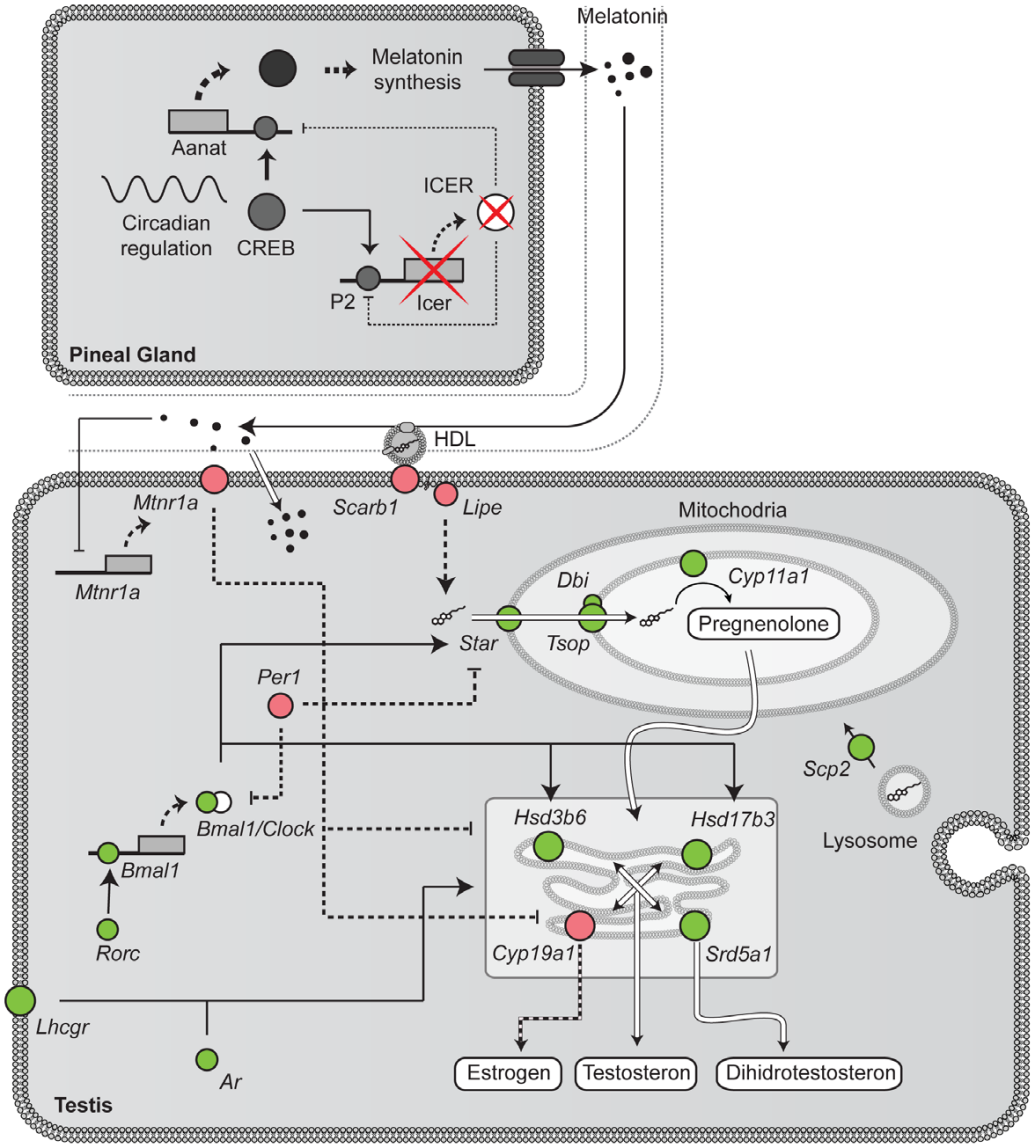
Steroidogenesis, melatonin and the circadian clock. Our data showed that several genes involved in steroid hormone synthesis (*Cyp11a1*, *Hsd17b3*, *Hsd3b6*, *Srd5a1*) and cholesterol transport (*Star*, *Tsop*, *Scp2*) are up-regulated in testes of *Crem* KO mice. On the other hand genes involved in the production of estrogens (*Cyp19a1*) and up-take of cholesterol (*Scarb1* and *Lipe*) are down-regulated. Many of these genes are under the control of both circadian factors (*Bmal1* and *Per1*) and the hormone melatonin. Melatonin can exert its effect either through the membrane receptor *Mtnr1a* or inside the cells through yet unresolved ways. Figure 6 also depicts the regulation of melatonin synthesis in the pineal gland. Here the main regulatory enzyme of melatonin synthesis *Aanat* is activated by phosphorylated CREB and inhibited by ICER, which in absent *Crem* KO animals.

The normal process of spermatogenesis is under tight hormonal regulation where follicle-stimulating hormone (FSH), luteinizing hormone (LH), testosterone and estrogens play important roles. FSH and LH do not exert their control directly on germ cells but rather regulate spermatogenesis through Sertoli and Leydig cell function. LH regulates testosterone production in Leydig cells which in turn together with FSH regulate Sertoli-germ cell interaction and signaling [42]–[47]. Several structural proteins as well as signaling pathways involved in cell-cell interactions are changed in KO testis. Even though plasma levels of testosterone do not differ between WT and KO mice (data not shown) a large number of enzymes, transporters and signaling molecules involved in steroidogenesis are altered in *Crem* KO testes (Table 3, Figure 6). An additional factor involved in normal spermatogenesis is also the circadian clock [48]. In testes of *Crem* KO mice *Bmal1*, *Per1*, *Cry1*, *Ror*-α, *CK1δ* and *CK1ε* were differentially expressed (Table 3) probably contributing to deregulation of steroidogenic enzymes. While most of the genes from steroid hormone synthesis (*Hsd17b3*, *Hsd3b6*, *Cyp11a1*, *Srd5a1*) and cholesterol trafficking (*Scp2*, *Star*, *Tsop*) are up-regulated, genes involved in cholesterol up-take (*Lipe*, *Scarb1*) were down-regulated (Figure 6). Additionally, *Cyp51* from cholesterol synthesis and *Cyp19* responsible for conversion of androgens to estrogens, both important for normal spermatogenesis [44], were down-regulated.

**Table 3:** Genes involved in cholesterol and androgen transport, synthesis and signal transduction.

Symbol	Gene Name	Gen ID	Function	Log 2 Fold Change
***enzymes***				
*Cyp51a1*	Lanosterol 14α demethylase	13121	converts lanosterol to cholesterol in the process of demethylation	−1.51
*Cyp39a1*	oxysterol 7-α-hydroxylase 2	56050	involved in the conversion of cholesterol to bile acids in peripheral tissues	−1.9
*Cyp19a1*	aromatase	13075	converts testosterone to estradiol	−0.72
*Cyp11a1*	Side-chain cleavage enzyme	13070	catalyzes the conversion of cholesterol to pregnenolone in three monooxygenase reactions	0.84
*Srd5a1*	5-alpha reductase	78925	catalyzes the conversion of testosterone into the more potent androgen, dihydrotestosterone (DHT)	0.59
*Hsd17b3*	17β-hydroxysteroid dehydrogenases	15487	catalyse the dehydrogenation of 17-hydroxysteroids in steroidogenesis	1.08
*Hsd3b6*	hydroxy-delta-5-steroid dehydrogenase	15497		1.25
*Sqle*	squalene epoxidase	20775	Catalyzes the first oxygenation step in sterol biosynthesis	0.75
***transporters***				
*Star*	steroidogenic acute regulatory protein	20845	regulates cholesterol transfer within the mitochondria	0.45
*Abca1*	cholesterol efflux regulatory protein	11303	cholesterol efflux pump in the cellular lipid removal pathway	0.89
*Scp2*	sterol carrier protein 2	20280	Participate in the intracellular transport of cholesterol and various other lipids	1.12
*Tsop*	translocator protein	12257	TSPO binds with high affinity to cholesterol and transports it across the mitochondrial membrane where it is used in steroid synthesis.	0.43
*Scarb1*	scavenger receptor class B, member 1	20778	Facilitates the flux of free and esterified cholesterol between the cell surface and extracellular donors and acceptors, such as HDL	−0.67
***receptors***				
*Ar*	androgen receptor	11835	nuclear receptor which is activated by binding of either of the androgenic hormones testosterone or dihydrotestosterone	0.79
*Lhcgr*	luteinizing hormone/choriogonadotropin receptor	16867	transmembrane receptor coupled to G-proteins that interacts with both luteinizing hormone (LH) and chorionic gonadotropins	0.78
***circadianrhythm***				
*Bmal1*	aryl hydrocarbon receptor nuclear translocator-like	11865	Core clock gene involved in the positive loop in circadian gene regulation	1.37
Per1	period homolog 1	18626	Core clock gene involved in the negative loop in circadian gene regulation	−0.5
Cry1	cryptochrome 1	12952	Core clock gene involved in the negative loop in circadian gene regulation	0.31
*Ck1δ*	casein kinase 1, delta	104318	Involved in Per and Cry phosphorylation - degradation	−0.34
*Rora*	RAR-related orphan receptor alpha	19883	Binds DNA as a monomer to hormone response elements (HRE) involved in circadian rhythm regulation	−2.07
*Rorc*	RAR-related orphan receptor gamma	19885	Binds DNA as a monomer to hormone response elements (HRE) involved in circadian rhythm regulation	1.12
*Rxra*	retinoid X receptor alpha	20181	RXR heterodimers act as ligand-dependent transcriptional regulators and increase the DNA-binding efficiency of its partner.	−1.24

## Discussion

The central role of *Crem* in spermatogenesis was confirmed simultaneously by two groups 15 years ago [15], [16]. Since then only one transcription profiling study was done on *Crem* KO mice by Beissbarth *et al* [8] in 2003. This study discovered a relatively small number of differentially expressed genes in testes from Crem KO mice indicating that only few genes are controlled by protein products of the *Crem* gene [8]. CREMτ is known to be a major transcriptional activator in post-meiotic germ cells [14], [23]. Thus, it is expected that the absence of CREM in *Crem* KO mice would have a profound effect on the global transcriptome of the testis. This assumption has been confirmed recently by CREM binding studies using ChIP-seq method, which revealed that the CREM protein could bind to more than 5000 genetic loci in testis [9]. In accordance to the ChIP-seq study, our data exposed 4706 DE genes.

The large discrepancy between ours and Beissbarth's data can in part be explained by the use of different microarray platforms. While Beissbarth *et al* used the MG U 74A microarrays that cover only about one third of the whole mouse genome, we used Affymetrix Mouse Gene 1.0 ST whole genome arrays. Many of genes expressed in haploid cells were not well characterized at the time of Beissbarth study [9]. Despite the different absolute numbers of differentially expressed genes, comparison of both microarray datasets shows a relatively large correlation (Figure 1). It is interesting to note that while Beissbarth found the majority of genes to be down-regulated, the opposite is seen in our data, with 2884 up- and 1822 down-regulated genes. Since CREM-τ is known as a potent activator highly expressed in round spermatids [14], [19], one would expect that the majority of genes in testes from *Crem* KO mice would be downregulated. However, because whole testes were used in the experiment, the up-regulation of genes can be explained by the presence of other somatic cells. One such example is the inducible cAMP early repressor ICER which is known to down-regulate the expression of *Creb* and *Fshr* gene in Sertoli cells [40], [41]. The lack of a functional ICER protein could thus explain the upregulation of both *Creb* and *Fshr* genes as confirmed by our data. However, it is important to take into account the possible up-regulation of these genes due to disrupted spermatogenesis in *Crem* KO mice. Since CREB is also known to regulate gene transcription in response to phosphorylation [49] the upregulation of *Creb* in KO testis can to some extent explain the relatively large number of upregulated genes in our study.

In order to determine which of DE genes are under direct CREM control we compared our dataset to the dataset from Martianov *et al*. where they applied chromatin immunoprecipitation of whole testis nuclei with the anti-CREM-τ antibody [14], [15], followed by sequencing of DNA fragments that bound the protein. Figures 2 and 3 show that 1607 (34.1%) DE genes from our data were reported to bind CREM. Of these, only 43% were down-regulated in testes of *Crem* KO mice. One reason for the low number of down-regulated genes might be that CREM binds to many more promoters than it actually regulates [9]. Additionally, the general transcriptional activity is highly up-regulated in haploid spermatids [50], [51], thus making euchromatin highly accessible to transcription factors. ChIP-seq experiment was similarly to our expression profiling conducted with the whole testes where other isoforms of *Crem* are present, among them repressor forms, which absence could lead to up-regulation of several genes [14], [19], [41]. Nevertheless, we discovered 698 genes whose expression was reduced in KO testis and could therefore be under direct regulation of CREM. The majority of other DE genes seem to be affected by secondary reasons of CREM absence. This is not unexpected since primary targets of CREM are transcriptional factors (Figure 3) that guide the subsequent transcriptional wave.

### Spermatogenesis

101 genes involved in spermatogenesis are DE in our dataset. Among them are several genes which were in recent years proven essential for normal sperm development (Table 2) [52]. We showed that all are down regulated in KO testis, with many of them having a high degree of expression change. *Crem* has been proven to regulate transcription of some of these genes, such as *Prm* 1 and 2 [53], *Tnp* 1 and 2 [30] and *Ace* [54] which were also shown to be bound by CREM in the experiment of Martianov *et al.* Additionally, we discovered several new genes (*Prm3*, *Tssk6*, *Pvrl2*, T*ekt5* and *Izumo1*) that are bound by the CREM protein in round spermatids and therefore potential novel targets of *Crem*. Interestingly, while *Prm3* was already discovered as down-regulated by Beissbarth *et al* that was not the case for *Prm* 1 and 2 [8].

### Apoptosis

As mentioned, *Crem* inactivation leads to increase in the rate of apoptosis at the stage of round spermatids [15], [16]. The expression of 258 genes related to apoptosis was changed in *Crem* KO testis. Of these almost half are bound by CREM (Figure 4). A proposed mechanism of apoptosis induction in male germ cells is presented in Figure 5, where DE genes from our data are colored according to their fold-change values. During normal spermatogenesis apoptosis occurs at several time points and is regulated by different pro- and anti-apoptotic molecules. At the stage of round spermatids *Fas* and *Fasl* expressed in germ and Sertoli cells, respectively, are the main regulators [55]. Up-regulation of *Fasl*, *NF-κB*, *IκB*, *Tnfr1* and several downstream targets of *Fas* indicate that round spermatid apoptosis is triggered *via* the extrinsic pathway (Figure 5). *Fasl* in Sertoli cells can be up-regulated indirectly by binding of TNFα, produced by germ cells, to the *Tnfr1* receptor expressed by Sertoli cells [56]. TNFα production in germ cell is linked to up-regulation of metalloproteinases, such as *Timp-1* [56], whose expression in Sertoli cells is regulated by FSH via FSH receptor (*Fshr*) [57]. Both *Fshr* and *Timp1* are up- regulated in KO testis. *Fas* receptor activation in germ cells induces expression of downstream signaling molecules such as caspase 8 or *Ask1* [58] to promote apoptosis (Figure 5). Similar to the *Aif1*, *BAD* also inhibits the anti-apoptotic protein *Bcl*-*2*. This inhibition is subject to the phosphorylation state of *BAD*, which is mediated by protein kinase A (PKA). Both *Akap1*, which targets *PKA* to mitochondria where BAD is located and phosphorylated as well as adenylate cyclase (*Ac*) and *PKA* are down regulated in KO testis [59]. Inhibition of *Bcl- 2* results in the activation of apoptosis inducing factor (*Aif*) and apoptosis. *Capn* and *Spna*, two proteins that were also implicated in apoptosis in germ cells, were also deregulated. Although fodrin is a substrate of calpain, calpain can also induce apoptosis through caspase 3/12 [60] (Figure 5).

Since these experiments were conducted on whole testis additional expression analysis using germ cells only would be necessary to provide definitive proof of above observations. However, since apoptosis in *Crem* deficient mice is elevated only in round spermatids [16], [61] these results could indicate how apoptosis might be induced and regulated in these cells.

### Melatonin, circadian clock and steroid metabolism

A functional circadian clock is essential for normal physiological functioning of animals. Perturbations of its components might lead to several diseases such as obesity [62], hypertension [63], breast cancer [64] and cardiovascular complications [65]. Both *Crem* and cAMP were shown to be important for the regulation of normal circadian rhythm [66], [67]. The first experiments in testis showed a total lack of a functional circadian clock [68], however the discovery that *Bmal1* is necessary for fertility and testosterone production in mice challenged this view [48]. The exact regulation of *Star* by *Bmal1* and other circadian factors is still not completely resolved [48]. Nevertheless, up-regulation of *Star*, *Hsd3b6* and *Hsd17b3* in our dataset could be a result of *Bmal1* up-regulation (Table 3 and Figure 6). *Bmal1* is known to be regulated by RAR-related orphan receptors (RORs) and in testis *Rorγt* is expressed at steady states. Since *Rorγt* is not under circadian control but could be regulated by transcription factors that bind E-boxes it could explain the up regulation of *Bmal1*. Furthermore, up-regulation of *StAR* could also be explained by the down-regulation of *Per1* [69]. *Per1* has previously been shown to be down regulated in *Crem* −/− mice [68] also confirming our results. It is important to note that the deregulated circadian components could be the consequence of melatonin synthesis, which is in *Crem* deficient mice up-regulated [70]. High levels of melatonin could repress steroidogenic genes in Leydig cells and thus lower testosterone which is crucial for spermatogenesis [71]. Melatonin inhibits estrogen biosynthesis by inhibiting aromatase activity at normal physiological levels [72], [73]. In our study, aromatase expression was repressed in KO testis while many other enzymes, transporters and signaling molecules that regulate testosterone production were up-regulated (Table 3). The reason for this could be that aromatase is expressed not only in Leydig cells but also in Sertoli and germ cells [74] and is therefore regulated in a different manners. Aromatase was repressed by *Icer* in ovarian granulosa cells and induced by 8-Br-cAMP in the Leydig cell line R2C [75] implying that it could also be under CREM control in germ cells.

The elevated expression of genes involved in testosterone production could indicate that intra-testicular levels of testosterone are lower than needed for normal spermatogenesis to occur. This would also explain the elevated expression of LH and androgen receptors and the down-regulated expression of melatonin receptor 1a. It is possible that due to the elevated synthesis of melatonin in *Crem* deficient mice during the night [76], the synthesis of testosterone is lower in KO than in normal mice. Thus, in the morning hours when our samples were taken (7:00 am) the reducing concentration of melatonin together with the down-regulation of *Mtnr1* would allow the up-regulation of testosterone synthesis stimulated by other factors. Although melatonin is supposed to act mainly through membrane receptors, it is capable of passing through plasma membranes and has been shown to interact directly with nuclear receptors and intracellular proteins [77]. In order to support our hypothesis that lower melatonin is responsible for up-regulated gene expression we additionally measured the expression of some metabolic and core clock genes at CT20 (4 hours prior to lights on) when the level of melatonin should be reaching its peak [78] (Supplementary Figure S4). Expression of *Rorc*, *Cyp11a1*, *Cyp17a1* and *Hsd17b3* are all significantly down-regulated indicating the effect of higher melatonin concentrations in CT20 compared to CT0. The trend of down-regulation (although not statistically significant) can also be observed for other genes (*Ar*, *Scarb1* and *Bmal1*), with the exception of *Srd5a*.

It is interesting to note that although many genes from steroid hormone synthesis are up-regulated, the uptake of cholesterol from HDL by *Scarb1* and the release of cholesterol from the plasma membrane by hormone sensitive lipase (*Lipe*) are both down-regulated. *Lipe* inhibition results in lowered steroidogenesis and has been shown to inhibit sperm production in testis [79].

Whether or not a functional circadian clock exists in the testis remains to be elucidated. We show that several clock genes are deregulated in CREM −/− animals and that this could be linked to deregulated melatonin and steroid synthesis. As already argued by Morse *et al* [68] the complex transcriptional process of spermatogenesis may not be compatible with the transcriptional process of circadian rhythms, however we still need to take into consideration the possible non-circadian function of clock proteins in this tissue.

### Cell junctions

**Figure 7:**
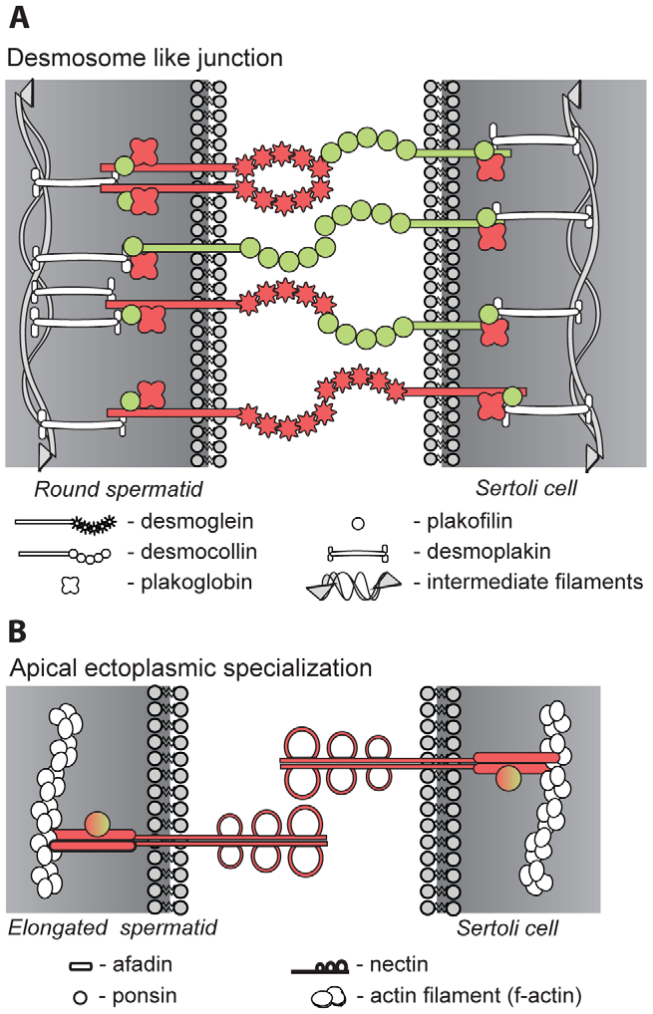
Cell-cell junctions. Two cell-cell junctions present between Sertoli and germ cells are presented: A - desmosome-like junctions and B - Ectoplasmic specialization. In DJ the down regulation of desmoglein and plakoglobin could lead to destabilization and separation of germ cells from Sertoli cells as seen by [16]. B - Apical EC are present between elongating or elongated spermatids and Sertoli cells. Down regulation of both germ and Sertoli specific nectin as well as its adaptor proteins that connect it to F-actin shows destabilization of the ES junction complex.

Normal spermatogenesis requires close contact between Sertoli and germ cells and between adjacent Sertoli cells. The Sertoli-germ cell junctions are maintained by desmosome-like junctions (DJ) and ectoplasmic specializations (ES) while a more elaborate contact, the blood – testis barrier (BTB), exists between the adjacent Sertoli cells [42]. 163 genes were DE in testis of KO mice (Figure 4) and seem to influence most of the junctions involved. The most negative effect of *Crem* absence is seen in DJ and to a lesser extent in ES (Figure 7). These are present between Sertoli and germ cells up to step VIII spermatids, and between Sertoli cells and elongating spermatids [42], [80]. Down-regulation of desmoglein (*Dsg1b*) and plakoglobin (*Jup*) proteins from DJ, and nectin (*Purl*) and afadin (*Mllt*) proteins present at ES would compromise the stability of germ cell adhesion and could explain the numerous round spermatids found in the lumen of *Crem* KO mice [16]. Importantly, both afadin and plakoglobin promoters bind CREM. On the other hand several proteins from the construction and maintenance of the BTB were up-regulated (N-cadherin (*Cdh2*), catenins (*Ctnn*), vinculin, *Tjp1* and *2*, integrin and *Jam2*). Similar results were also seen when intratesticular levels of testosterone were reduced, which resulted in the destruction of junction integrity at both apical ES and non-ES junctions while strengthening the function of the BTB [81], [82]. Up-regulation of *RhoB*, a small GTPase involved in actin reorganization [47] indicates disruptions at the apical ES, since its up-regulation is also seen in drug induced ES disruptions [47], [83]. Several other regulatory proteins from actin and microtubule dynamics were also deregulated in KO testis (Table 4).

**Table 4:** Proteins affecting actin and microtubule dynamics in testis.

Symbol	Expression	Function	Fold-change	Bound by *CREM*
*Wasf2*	n.d.	Actin polymerization	−1.38	Yes
*RhoB*	SC>GC	Regulation of Sertili germ cell adhesion	0.44	Yes
*Rac1*	n.d.	n.d.	0.27	No
*Cdc42*	n.d.	n.d.	0.42	Yes
*Tesk2*	SC	n.d.	0.65	No
*ArpM1*	spermatid	GC morphogenesis	−1.19	Yes
*Actl7a*	spermatid	GC morphogenesis/sperm function	−3.81	No
*Actrl1*	spermatozooa	GC morphogenesis/sperm function	−3.29	No
*Actrl2*	spermatozooa	GC morphogenesis/sperm function	−5.22	Yes
*Clip1*	spermatid	Spermatid differentiation	−2.26	Yes
*Ptk2*	BTB	Restructuring at BTB	0.47	Yes

In conclusion, our large scale expression analysis study in which whole testis of *Crem* deficient mice were analyzed showed that the absence of *Crem* has a larger impact on different aspects of spermatogenesis as estimated previously. These changes range from apoptosis induction to deregulation of major circadian clock genes, steroidogenesis and the cell-cell junction dynamics. Several new genes important for normal spermatogenesis and fertility were shown to be strongly down-regulated in KO testis and are possible novel targets of CREM. Proteomic and metabolomic analysis would enable a more detailed prediction and elucidation of physiological roles of CREM in steroidogenesis and other signaling and metabolic pathways. Additionally, cell specific knock-out models of mice would provide a clearer insight into the cell-specific functions of *Crem*.

## Materials and Methods

### Animals

Nine 8 weeks old WT and *Crem* KO male mice and five 30 days old WT and *Crem* KO pre-pubertal male mice of the mixed strain 129S2/SvPasCrlf×C57BL/6JRj [15] were maintained in a temperature and humidity controlled room under a 12∶12 h light∶dark cycle (light on at 7:00 am, light off at 7:00 pm) with free access to food (Harlan Teklad 2916) and acidified water (pH = 3). Genotyping results done by PCR are presented in Figure S5. The experiment was approved by the Veterinary Administration of the Republic of Slovenia (license number 34401-9/2008/4) and was conducted in accordance with the European Convention for the protection of vertebrate animals used for experimental and other scientific purposes (ETS 123) as well as in accordance with National Institutes of Health guidelines for work with laboratory animals.

### RNA extraction

Mice were sacrificed with cervical dislocation. Tissue samples, including testes, were excised, snap frozen in liquid nitrogen and stored at −80°C for subsequent analysis. Whole testes were first pulverized and then homogenized in 1000 µl of TRI Reagent (Sigma) and total RNA was isolated according to the manufacturer's instructions. RNA quantity and quality (Table S6) were assessed with NanoDrop and Agilent 2100 Bioanalyzer instruments, all samples isolated fulfilled the minimum requirements (OD_260/280_ and OD_230/280_>1.8) for microarray hybridization.

### cDNA preparation and qPCR

cDNA was prepared for validation of microarray data using quantitative real-time PCR. DNAse treatment was performed on all samples using DNAse I (Roche Applied Bioscience) according to the manufacturer's instructions. cDNA synthesis was carried out using SuperScript III reverse transcriptase (Invitrogen). 1 µg of RNA was mixed together with 20 µl of reverse transcriptase master mix which contained 8 µl of 5× first strand buffer, 2 µl of 100 mM DTT, 2 µl of 10 mM dNTP mix, 1 µl of random primers (Promega 500 ng/µl), 0.75 µl of SuperScript III (200 U/µl), 0.75 µl of RNAse OUT (Invitrogen) and 5.5 µl of RNAse free water in a final volume of 40 µl. The reaction mixtures were incubated at 25°C for 5 minutes, 50°C for 60 minutes and 70°C for 10 minutes. Real time quantitative PCR was performed in a 384 well format on LightCycler 480 (Roche Applied Science) using LightCycler 480 SYBR Green I Master (Roche Applied Science). The PCR reaction consisted of 2.5 µl of SYBR Green I Master, 1.15 µl of RNAse free water, 0.6 µl of 300 nM primer mix and 0.75 µl of cDNA in a total volume of 5 µl. Three technical replicates were performed for each sample. Cycling conditions were as follows: 10 minutes at 95°C followed by 40 rounds of 10 s at 95°C, 20 s at 60°C and 20 s at 72°C. Melting curve analysis for determining the dissociation of PCR products was preformed from 65°C to 95°C.

### Primer design and qPCR normalization

Wherever possible, intron spanning primers were designed based on publicly available sequences. Primer specificity and amplification efficiency were validated empirically with melting curve and standard curve analysis of a six fold dilution series respectively. Primer information is reported in Table S7. Reference gene selection and normalization was done according to [84] with the help of Eq-PCR Wizzard software.

### Microarray hybridization

100 ng of total RNA was used for hybridization of Affymetrix Gene Chip Mouse 1.0 ST Arrays according to manufacturer's instructions (Genechip® Whole Transcript (wt) Sense Target Labeling Assay Manual – P/N 701880 Rev 4). In brief: total RNA was used to synthesize double-stranded cDNA with random hexamer primers tagged with a T7 promoter sequence. The double-stranded cDNA was subsequently used as a template and amplified by T7 RNA polymerase producing cRNA. In the second cycle of cDNA synthesis, random hexamers were used to prime reverse transcription of the cRNA and dUTP was incorporated to produce single-stranded DNA. This single-stranded DNA sample were then treated with a combination of uracil DNA glycosylase (UDG) and apurinic/apyrimidinic endonuclease 1 (APE 1) that specifically recognized the unnatural dUTP residues and broke the DNA strand. DNA was labeled by terminal eoxynucleotidyl transferase (TdT) with the Affymetrix proprietary DNA Labeling Reagent that is covalently linked to biotin. 10 samples (5 wild-type and 5 knock-out) were labeled as described, injected into Affymetrix arrays and hybridized at 45°C and 60 rpm for 16 h in an Affymetrix Hybridization Oven. Arrays were later stained and washed in the Affymetrix GeneChip Fluidic Station 450 and scanned using Affymetrix GeneChip Scanner 3000 7G. Preliminary assessment of the data generated was made using Expression Console (Affymetrix).

### Microarray data analysis

GeneChip data was processed and analyzed using R/Bioconductor packages. Data were normalized using RMA algorithm from XPS package (based on core meta-probeset annotations and AFFX control probes, by grouping probes to the level of transcripts and computing background from antigenomic probes). The raw and normalized gene expression data together with experimental information are deposited in Gene Expression Omnibus database (http://www.ncbi.nlm.nih.gov/geo, accession number GSE29593) in compliance with MIAME standards [85].

Differential expression of genes between the wild type and *Crem* knockout mice testes samples was assessed using LIMMA package by controlling the false discovery rate [86] at significance level α = 0.05. Annotation of genes and data representation was managed using ANNAFFY and AFFYCORETOOLS packages. In parallel, gene set enrichment analysis was performed using Parametric Gene Set Enrichment Analysis (PGSEA) package in combination with the LIMMA package by controlling the false discovery rate at significance level α = 0.05. Gene sets related to different KEGG pathways and GO-BP (Gene Ontology – Biological Process) terms were tested for their enrichment between the two mice samples and sorted by their enrichment scores.

Venn and Euler diagrams (package VENNEULER) were used to cross-compare the lists of DE genes with other experimental data and data on transcription factors by partition corresponding DE genes into Venn regions.

## Supporting Information

Figure S1 **Crem gene locus structure, its transcripts and** ***Crem*** **KO construct.** Crem isoforms are transcribed from either the P1 promoter, producing *Crem* activators or repressors or from the internal cAMP responsive P2 promoter producing *Icer* repressors. The *Crem* KO construct was produced by insertion of a neomycine resistance gene between the *SmaI* and *SalI* sites thereby straddling the two alternative DNA binding domains (DBD I and DBD II) [15]. Q1 and Q2 - kinaze inducible domains; P-box - phosphorylation box; ATG – translation start codon; TTA and TAG – STOP codons.
(TIF)

Figure S2 **Multidimensional scaling.** Multidimensional scaling of normalized data exposed clear separation between wild-type and knockout animals. MDS was done for the top 1038 genes. A similar separation is also seen when using lower number of genes.
(PDF)

Figure S3 **Expression of gene in prepubertal mice.** Expression of key genes was measured by qPCR in prepubertal mice (30 days old) in order to confirm that down-regulation is not a consequence of the absence of particular cells *in Crem* KO mice. It is know that cells from stage 4 round spermatids onward are missing in Crem KO mice due to apoptosis of round spermatids. All genes shown have a statistically significant down regulation in KO animals in both adult and pre-pubertal mice.
(PDF)

Figure S4 Expression of metabolic (*Cyp11a1*, *Cyp17*, *Hsd17b3. Srd5a*, *Scarb1* and *Ar*) and core clock genes (*Bmal1*, *Per1*, *Rorc*) was measured at time points CT0 and CT20. A statistically significant down regulation of *Cyp11a1*, *Cyp17*, *Hsd17b3* and *Rorc* was seen in CT20 compared to CT0. This confirms that the elevated levels of melatonin at CT20 repress expression of selected genes in testis. *Bmal1*, *Per1* and *Ar* also show a trend to be down regulated in CT20 however this was not statistically significant.
(PDF)

Figure S5 **Genotyping of animals.** Animals were genotyped to ensure that the proper ones were chosen for the study. Genotyping is done using a standard PCR protocol and separation of the product on a 2% agarose gel. Wild-type fragment is 524 bp in length while the knockout fragment is only 406 bp long. A clear separation can be seen between wild-type and knockout animals.
(TIF)

Table S1 Differentially expressed genes as detected by GeneChip Mouse Gene 1.0 ST Affymetrix microarrays between *Crem* knockout and wild-type mice testis.
(XLS)

Table S2 Differentially expressed genes from Beissbarth *et al.* 2003 data using our analysis methods and conditions.
(XLS)

Table S3 A list of genes found in the cross-section between DE genes from Beissbarth *et al.* dataset and DE genes from Kosir *et al.* dataset.
(XLS)

Table S4 A list of genes found in the cross-section between DE genes from Kosir *et al.* and genes bound by CREM in testis from Martianov *et al.*
(XLS)

Table S5 A list of genes found in the cross-section between DE genes from Kosir *et al.* and the TFCat database from Fulton *et al*.
(XLS)

Table S6 RNA quantity and quality as measured by NanoDrop and Agilent 2100 Bioanalyzer.
(DOC)

Table S7 Information for primers used in the validation of microarray data by qPCR.
(DOC)
