# Supplementary figures and images for: Novel Insights into the Downstream Pathways and Targets Controlled by Transcription Factors CREM in the Testis
Source: PLoS One. 2012 Feb 22;7(2):e31798. doi: 10.1371/journal.pone.0031798 (PMC3285179; doi:10.1371/journal.pone.0031798)

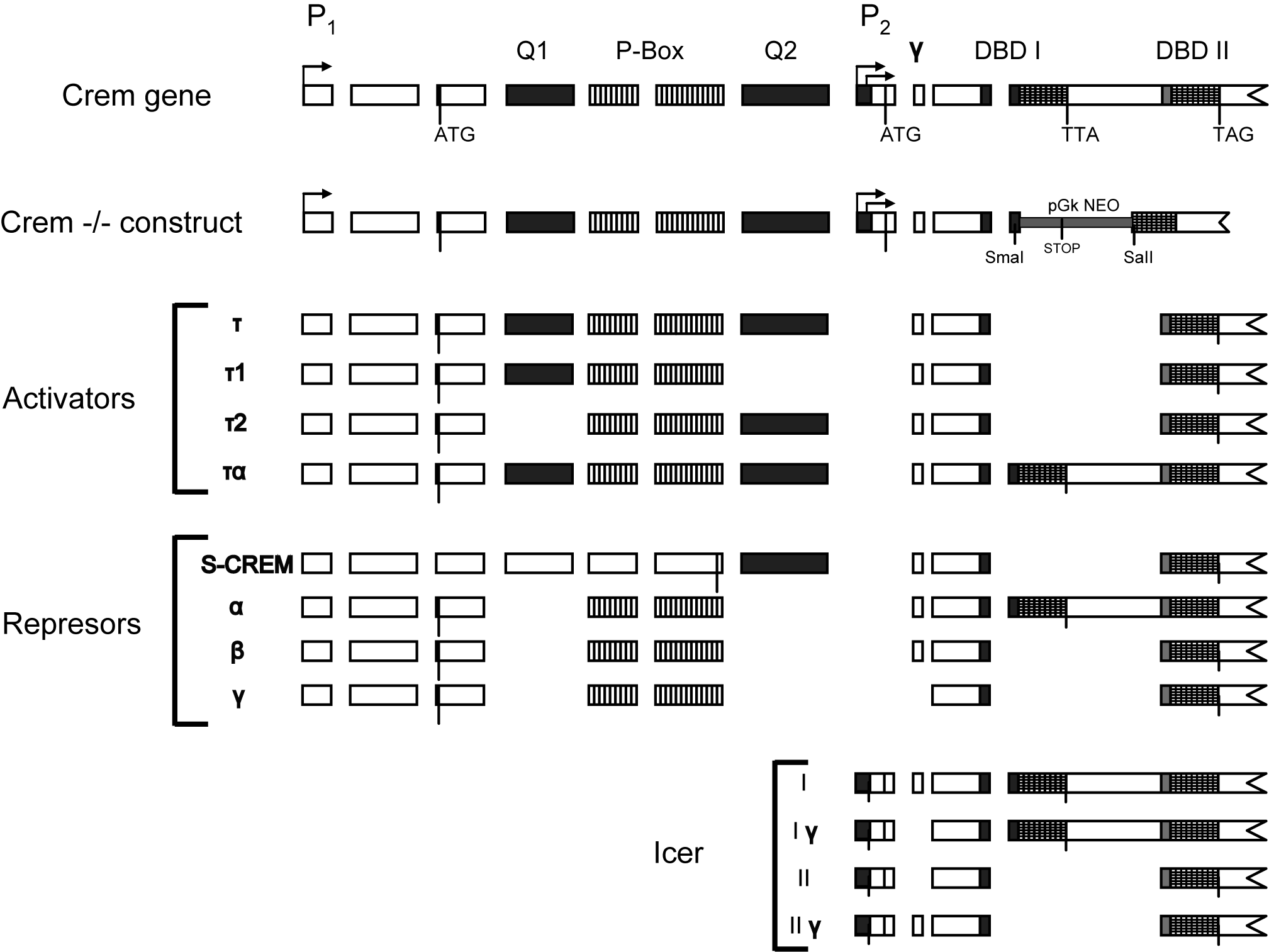

Supplement: Figure S1 — Crem gene locus structure, its transcripts and Crem KO construct. Crem isoforms are transcribed from either the P1 promoter, producing Crem activators or repressors or from the internal cAMP responsive P2 promoter producing Icer repressors. The Crem KO construct was produced by insertion of a neomycine resistance gene between the SmaI and SalI sites thereby straddling the two alternative DNA binding domains (DBD I and DBD II) [15]. Q1 and Q2 - kinaze inducible domains; P-box - phosphorylation box; ATG – translation start codon; TTA and TAG – STOP codons. (TIF) [file pone.0031798.s001.tif]

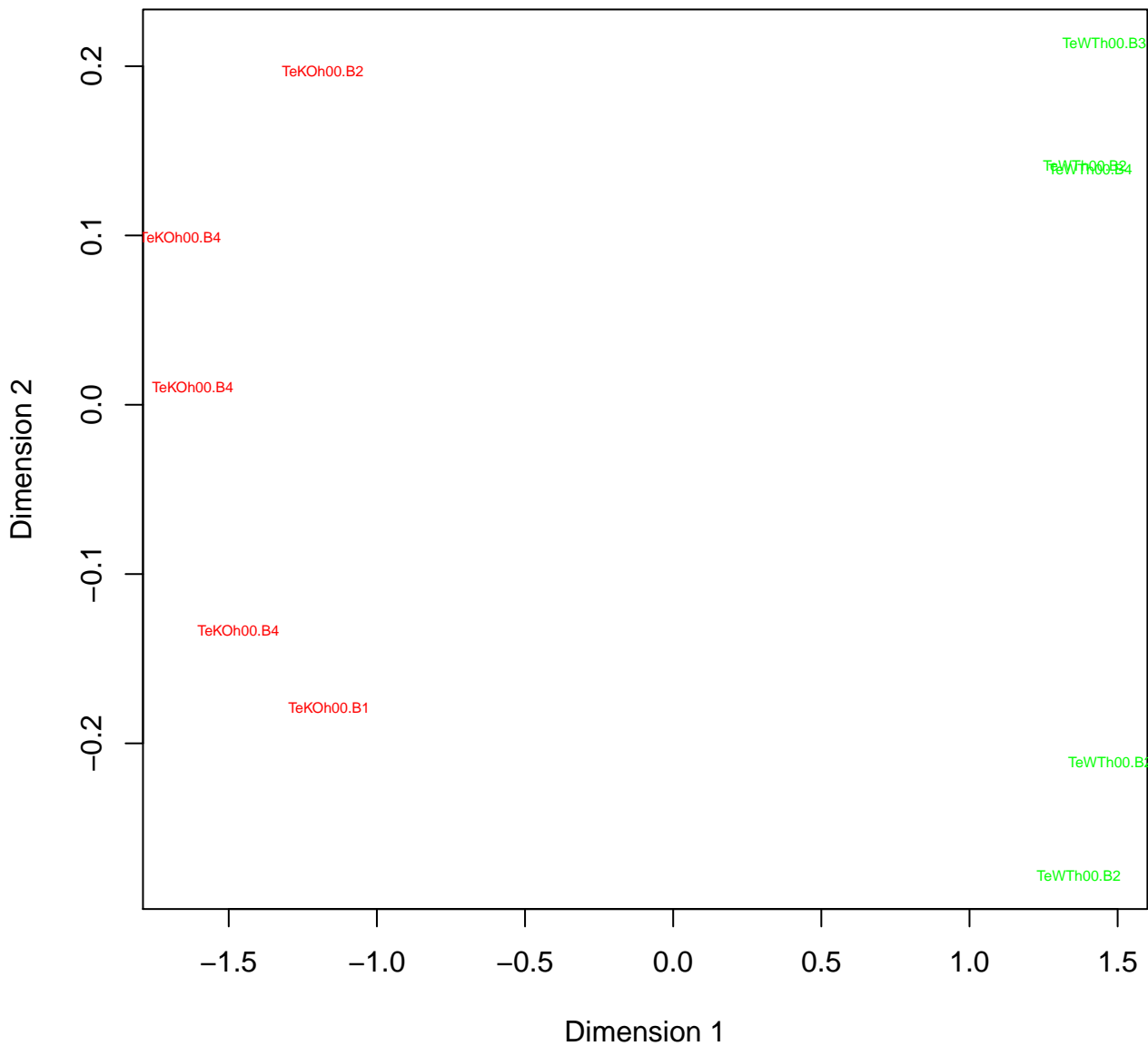

Supplement: Figure S2 — Multidimensional scaling. Multidimensional scaling of normalized data exposed clear separation between wild-type and knockout animals. MDS was done for the top 1038 genes. A similar separation is also seen when using lower number of genes. (PDF) [file pone.0031798.s002.pdf]

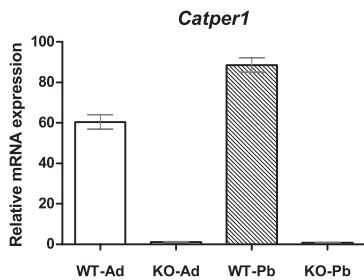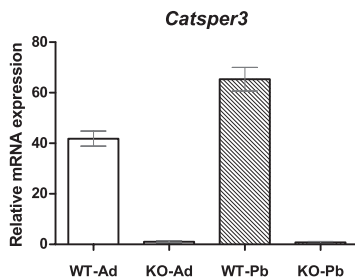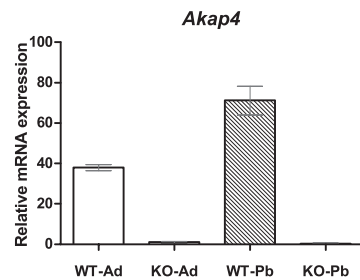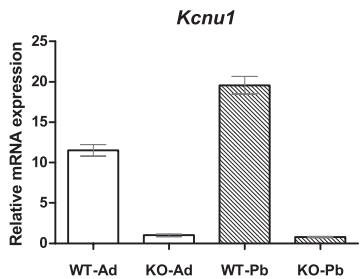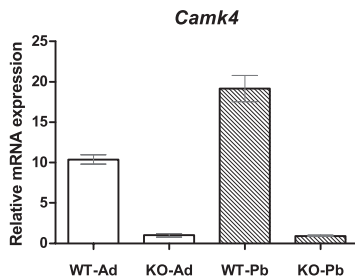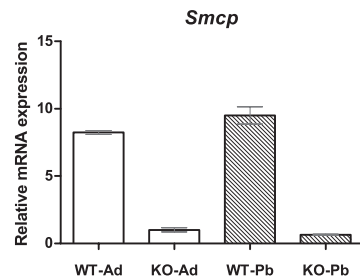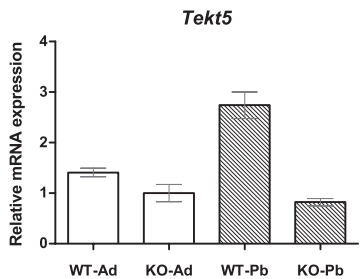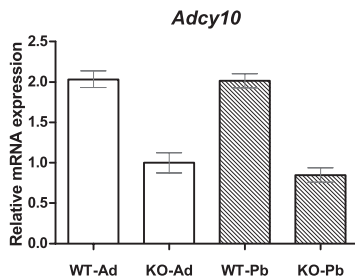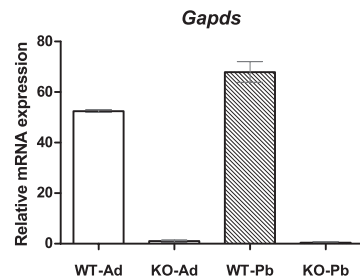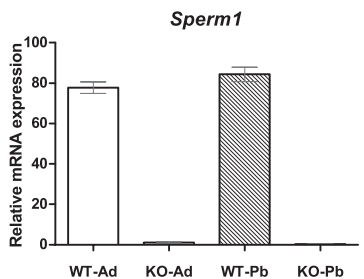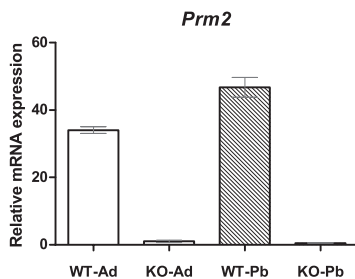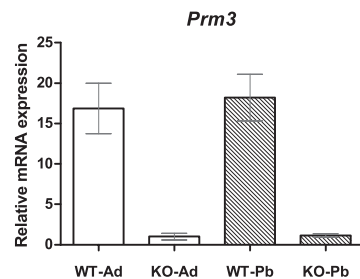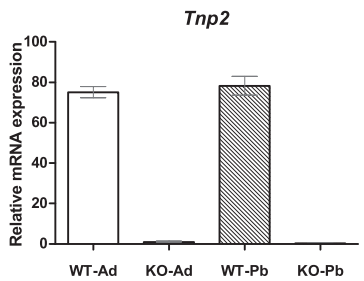

Supplement: Figure S3 — Expression of gene in prepubertal mice. Expression of key genes was measured by qPCR in prepubertal mice (30 days old) in order to confirm that down-regulation is not a consequence of the absence of particular cells in Crem KO mice. It is know that cells from stage 4 round spermatids onward are missing in Crem KO mice due to apoptosis of round spermatids. All genes shown have a statistically significant down regulation in KO animals in both adult and pre-pubertal mice. (PDF) [file pone.0031798.s003.pdf]

*Bmal1*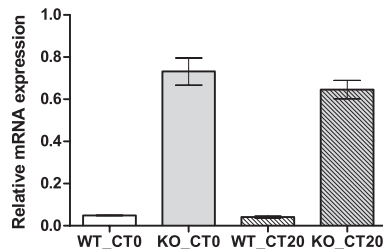*Per1*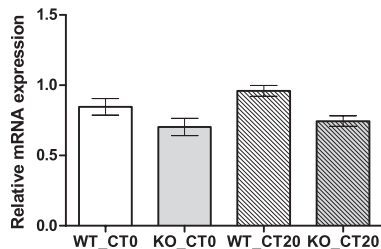*Rorc*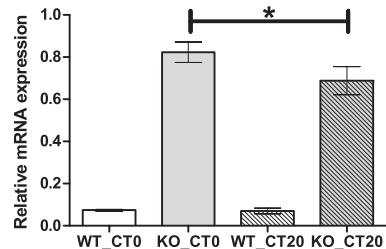*Cyp11a1*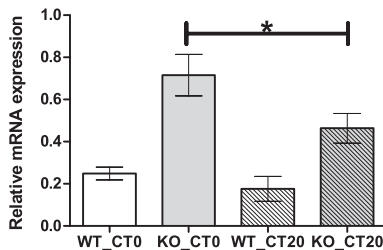*Cyp17*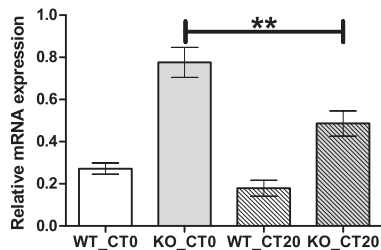*Hsd17*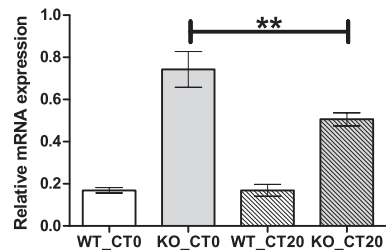*Srd5a*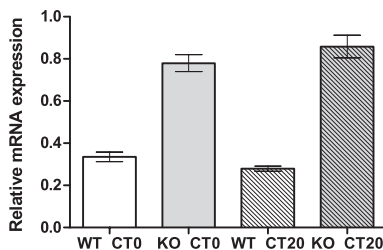*Scarb1*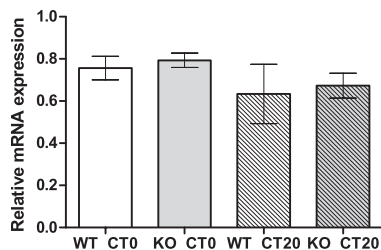*Ar*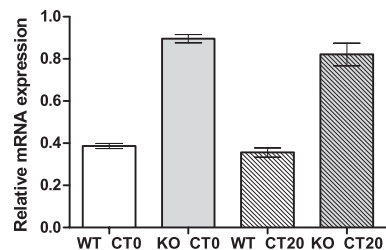

Supplement: Figure S4 — Expression of metabolic (Cyp11a1, Cyp17, Hsd17b3. Srd5a, Scarb1 and Ar) and core clock genes (Bmal1, Per1, Rorc) was measured at time points CT0 and CT20. A statistically significant down regulation of Cyp11a1, Cyp17, Hsd17b3 and Rorc was seen in CT20 compared to CT0. This confirms that the elevated levels of melatonin at CT20 repress expression of selected genes in testis. Bmal1, Per1 and Ar also show a trend to be down regulated in CT20 however this was not statistically significant. (PDF) [file pone.0031798.s004.pdf]

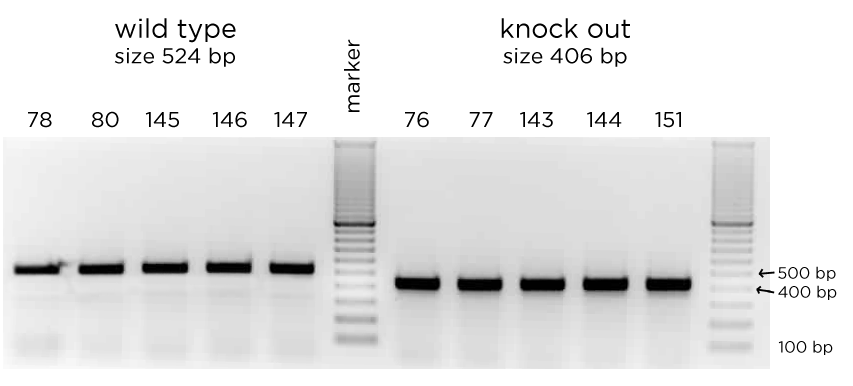

Supplement: Figure S5 — Genotyping of animals. Animals were genotyped to ensure that the proper ones were chosen for the study. Genotyping is done using a standard PCR protocol and separation of the product on a 2% agarose gel. Wild-type fragment is 524 bp in length while the knockout fragment is only 406 bp long. A clear separation can be seen between wild-type and knockout animals. (TIF) [file pone.0031798.s005.tif]
