# Supplementary material for: Novel Insights into the Downstream Pathways and Targets Controlled by Transcription Factors CREM in the Testis
Source: PLoS One. 2012 Feb 22;7(2):e31798. doi: 10.1371/journal.pone.0031798 (PMC3285179; doi:10.1371/journal.pone.0031798)
Supplement: Table S6 — RNA quantity and quality as measured by NanoDrop and Agilent 2100 Bioanalyzer. (DOC) [file pone.0031798.s011.doc]

**Table S6:** RNA quantity and quality as measured by NanoDrop and Agilent 2100 Bioanalyzer.

| **Number** | **Genotype** | | **Mouse ID** | | **NanoDrop** | | | | | | **Agilent** |
| --- | --- | --- | --- | --- | --- | --- | --- | --- | --- | --- | --- |
|  |  |  |  |  | **Concentration [ng/ul]** | | **260/280 ratio** | | **260/230 ration** | | **RIN** |
| **Adult mice** | |  | |  | |  | |  | |  | |
| 1 | K | | 76 | | 1260.96 | | 1.98 | | 2.35 | | 9.1 |
| 2 | K | | 77 | | 1288.7 | | 1.98 | | 2.28 | | 9.1 |
| 3 | K | | 143 | | 1301.62 | | 1.97 | | 2.35 | | 9.3 |
| 4 | K | | 144 | | 1401.63 | | 1.98 | | 2.13 | | 9.2 |
| 5 | K | | 151 | | 1460.92 | | 1.99 | | 2.26 | | 9.1 |
| 6 | W | | 78 | | 1760.06 | | 1.99 | | 2.29 | | 8.9 |
| 7 | W | | 80 | | 1574.73 | | 1.99 | | 2.3 | | 8.8 |
| 8 | W | | 145 | | 2044.66 | | 2.01 | | 2.25 | | 8.9 |
| 9 | W | | 146 | | 1989.73 | | 2 | | 2.27 | | 8.8 |
| 10 | W | | 147 | | 1857.69 | | 2 | | 2.27 | | 8.8 |
| 11 | K | | 124 | | 835.31 | | 1.97 | | 2.38 | | N.D. |
| 12 | K | | 125 | | 828.78 | | 1.97 | | 2.37 | | N.D. |
| 13 | K | | 186 | | 1076.43 | | 1.97 | | 2.35 | | N.D. |
| 14 | K | | 187 | | 843.29 | | 1.96 | | 2.36 | | N.D. |
| 15 | W | | 188 | | 1140.48 | | 1.97 | | 2.34 | | N.D. |
| 16 | W | | 189 | | 1188.25 | | 1.99 | | 2.28 | | N.D. |
| 17 | W | | 190 | | 1057.08 | | 2.00 | | 2.26 | | N.D. |
| 18 | W | | 191 | | 1106.34 | | 2.02 | | 2.18 | | N.D. |
| **Pre-pubertal mice** | |  | |  | |  | |  | |  | |
| 1 | W | | 1 | | 1219.64 | | 2.03 | | 2.37 | | 9.2 |
| 2 | W | | 2 | | 932.5 | | 2.01 | | 2.4 | | 9.4 |
| 3 | W | | 3 | | 1306.17 | | 2.02 | | 2.35 | | 9.2 |
| 4 | W | | 4 | | 1350.73 | | 2.03 | | 2.35 | | 9.4 |
| 5 | W | | 5 | | 1196.49 | | 2.02 | | 2.37 | | 9.3 |
| 6 | K | | 6 | | 822.82 | | 1.99 | | 2.39 | | 9.6 |
| 7 | K | | 7 | | 830.54 | | 2.01 | | 2.39 | | 9.7 |
| 8 | K | | 8 | | 1023.45 | | 2 | | 2.38 | | 9.4 |
| 9 | K | | 9 | | 1065.97 | | 2.03 | | 2.37 | | 9.7 |
| 10 | K | | 10 | | 1073.73 | | 2.02 | | 2.37 | | 9.7 |
