# Supplementary material for: Novel Insights into the Downstream Pathways and Targets Controlled by Transcription Factors CREM in the Testis
Source: PLoS One. 2012 Feb 22;7(2):e31798. doi: 10.1371/journal.pone.0031798 (PMC3285179; doi:10.1371/journal.pone.0031798)
Supplement: Table S7 — Information for primers used in the validation of microarray data by qPCR. (DOC) [file pone.0031798.s012.doc]

**Table S7: Information for primers used in qPCR validation of micro array data.**

| **Gene** | **NCBI ID** |  | **Sequence** | **Lenght of primer in [bp]** | **Efficiency** |
| --- | --- | --- | --- | --- | --- |
| ***Reference genes*** | |  |  |  |  |
| *Rplp0* | NM_007475.4 | fw | *CACTGGTCTAGGACCCGAGAAG* | 22 | 1,98 |
|  |  | rv | *GGTGCCTCTGGAGATTTTCG* | 20 |  |
| *Ppib* | NM_011149.2 | fw | *GGAGATGGCACAGGAGGAAA* | 20 | 1,93 |
|  |  | rv | *CCGTAGTGCTTCAGTTTGAAGTTCT* | 25 |  |
| *Gapdh* | NM_008084.2 | fw | *CCAATGTGTCCGTCGTGGATCT* | 22 | 1,94 |
|  |  | rv | *GTTGAAGTCGCAGGAGACAACC* | 22 |  |
| *Actb* | NM_007393.3 | fw | *CTTCCTCCCTGGAGAAGAGC* | 20 | 1,98 |
|  |  | rv | *ATGCCACAGGATTCCATACC* | 20 |  |
| *Hmbs* | NM_013551.2 | fw | *TCCCTGAAGGATGTGCCTA* | 19 | 1,64 |
|  |  | rv | *AAGGGTTTTCCCGTTTGC* | 18 |  |
| *Hprt* | NM_013556.2 | fw | *TCCTCCTCAGACCGCTTTT* | 19 | 1,89 |
|  |  | rv | *CCTGGTTCATCATCGCTAATC* | 21 |  |
| *Rn18s* | NR_003278.1 | fw | *CGCCGCTAGAGGTGAAATTC* | 20 | 1,79 |
|  |  | rv | *TTGGCAAATGCTTTCGCTC* | 19 |  |
| *Eif2a* | NM_001005509.1 | fw | *CAACGTGGCAGCCTTACA* | 18 | 1,95 |
|  |  | rv | *TTTCATGTCATAAAGTTGTAGGTTAGG* | 27 |  |
| *Utp6* | NM_144826.3 | fw | *TTTCGGTTGAGTTTTTCAGGA* | 21 | 1,82 |
|  |  | rv | *CCCTCAGGTTTACCATCTTGC* | 21 |  |
| *Tbcc* | NM_178385.3 | fw | *GACTCCTTCCTGAACCTCTGG* | 21 | 1,90 |
|  |  | rv | *GGAGGCCATTCAAAACTTCA* | 20 |  |
| ***Circadian genes*** | |  |  |  |  |
| *Bmal1* | NM_007489 | fw | *GCAGTGCCACTGACTACCAAGA* | 22 | 1,94 |
|  |  | rv | *TCCTGGACATTGCATTGCAT* | 20 |  |
| *Per1* | NM_011065.3 | fw | *TCCTCCTCCTACACTGCCTCT* | 21 | 1,90 |
|  |  | rv | *TTGCTGACGACGGATCTTT* | 19 |  |
| *Cry1* | NM_007771 | fw | *CCCAGGCTTTTCAAGGAATGGAACA* | 25 | 1,98 |
|  |  | rv | *TCTCATCATGGTCATCAGACAGAGG* | 25 |  |
| ***Metabolic genes*** | |  |  |  |  |
| *Rxra* | NM_011305.3 | fw | *GCTTCGGGACTGGTAGCC* | 18 | 1,99 |
|  |  | rv | *GCGGCTTGATATCCTCAGTG* | 20 |  |
| *Cyp51* | NM_020010.2 | fw | *ACGCTGCCTGGCTATTGC* | 18 | 1,86 |
|  |  | rv | *TTGATCTCTCGATGGGCTCTATC* | 23 |  |
| *Cyp11a1* | NM_019779.3 | fw | *AAGTATGGCCCCATTTACAGG* | 21 | 1,99 |
|  |  | rv | *TGGGGTCCACGATGTAAACT* | 20 |  |
| *Cyp39a1* | NM_018887.3 | fw | *ACCTATGATGAGGGCTTTGAGTA* | 23 | 1,84 |
|  |  | rv | *CCATCTTTTGGATTTTGACCA* | 21 |  |
| ***Rorc*** | NM_011281.2 | fw | *ACCTCTTTTCACGGGAGGA* | 20 | 1,93 |
|  |  | rv | *TCCCACATCTCCCACATTG* | 20 |  |
| *Hsd17b3* | NM_008291.3 | fw | *AATATGTCACGATCGGAGCTG* | 21 | 1,85 |
|  |  | rv | *GAAGGGATCCGGTTCAGAAT* | 20 |  |
| *Sqle* | NM_010191.2 | fw | *CCAAACAGGACTGGGACAAG* | 21 | 1,92 |
|  |  | rv | *GACGAGAAAGGCCAATTCC* | 20 |  |
| ***Other*** |  |  |  |  |  |
| *Scarb1* | NM_016741.1 | fw | *GCCCATCATCTGCCAACT* | 18 | 1,89 |
|  |  | rv | *TCCTGGGAGCCCTTTTTACT* | 20 |  |
| *Ace* | NM_207624.4 | fw | *TCTGCTTCCCCAACAAGACT* | 20 | 1,85 |
|  |  | rv | *AGGATGTTGGTGAGCTCTGG* | 20 |  |
| *Ar* | NM_013476.3 | fw | *CCAGTCCCAATTGTGTCAAA* | 20 | 1,79 |
|  |  | rv | *TCCCTGGTACTGTCCAAACG* | 20 |  |
| *Fshr* | NM_013523.3 | fw | *ACCCTGAGGCCTTCCAGA* | 18 | 1,87 |
|  |  | rv | *AGTGTTTAATGCCTGTGTTGGA* | 22 |  |
| *Scp2* | NM_011327.3 | fw | *TGCGTTGGCTATGTGTATGG* | 18 | 1,82 |
|  |  | rv | *TGCCAGTCAGTCCCAAACTA* | 22 |  |
| Arnt | NM_001037737.2 | fw | tgcctcatctggtactgctg | 20 | 1,98 |
|  |  | rv | tgtcctgtggtctgtccagt | 20 |  |
| Atf7ip | NM_019426.2 | fw | ccaccatcctttcaaactcc | 20 | 1,94 |
|  |  | rv | ctgctgtagaagtggcgtga | 20 |  |
| Atoh8 | NM_153778.3 | fw | tcagcttctccgagtgtgtg | 20 | 1,95 |
|  |  | rv | tagcctgtggcaggtcact | 19 |  |
| Bach2 | NM_001109661.1 | fw | cagtgagtcgtgtcctgtgc | 20 | 1,92 |
|  |  | rv | ttcctgggaaggtctgtgat | 20 |  |
| Cbfa2t3 | NM_009824.2 | fw | tggctacatgcctgaagagat | 21 | 2 |
|  |  | rv | gcgcttcacctcattcaca | 19 |  |
| Ctnnb1 | NM_007614.3 | fw | gcagcagcagtttgtgga | 18 | 1,55 |
|  |  | rv | tgtggagagctccagtacacc | 21 |  |
| Dlx6 | NM_010057.2 | fw | ccatgcagagaccacagatg | 20 | 1,99 |
|  |  | rv | aatgctgccatgtttgtgc | 19 |  |
| Eid2 | NM_198425.2 | fw | atgccgtacatgcgcttc | 18 | 1,94 |
|  |  | rv | tcgggtagttctccaggtagtg | 22 |  |
| Hnrnpab | NM_010448.3 | fw | gcaacagcagtatggctctg | 20 | 1,94 |
|  |  | rv | ccgtaatttgtactaccctgacct | 24 |  |
| Mllt11 | NM_019914.4 | fw | tcggaacaccctagtcattca | 21 | 1,87 |
|  |  | rv | atgggcatcctccagaaaa | 19 |  |
| Mllt3 | NM_027326.3 | fw | gacactgagggaacgacaca | 20 | 1,92 |
|  |  | rv | tgtccagtttcctctataaggttca | 25 |  |
| Ncoa3 | NM_008679.3 | fw | ctggcactgctgtgatgag | 19 | 1,86 |
|  |  | rv | agccatttgggcattaaaga | 20 |  |
| Nfe2l1 | NM_008686.3 | fw | cgacagactgtgacctcagc | 20 | 1,72 |
|  |  | rv | caggtctttgtcttgggtcag | 21 |  |
| Nr4a1 | NM_010444.2 | fw | ctgtccgctctggtcctc | 18 | 2 |
|  |  | rv | aatgcgattctgcagctctt | 20 |  |
| Pax5 | NM_008782.2 | fw | acgctgacagggatggtg | 18 | 1,99 |
|  |  | rv | ggggaacctccaagaatcat | 20 |  |
| Paxip1 | NM_018878.2 | fw | aaaccttaacaagaagtgcacaca | 24 | 1,9 |
|  |  | rv | tgacagcacgctcgtatttc | 20 |  |
| Ring1 | NM_009066.3 | fw | caaaacgtgggaactgagtct | 21 | 1,91 |
|  |  | rv | cgcaatctctgtaccatcca | 20 |  |
| Sall3 | NM_178280.3 | fw | ccaagggcaatctcaaggt | 19 | 2 |
|  |  | rv | agagccatggggttctcc | 18 |  |
| Scrt1 | NM_130893.3 | fw | cattctcttcggcagacctc | 20 | 1,98 |
|  |  | rv | ccacgtagtcactgaggtatcctt | 24 |  |
| Sin3a | NM_001110351.1 | fw | tggaagcagaggtgtggac | 19 | 1,91 |
|  |  | rv | tgtgccagatgttctcgaag | 20 |  |
| Smarcc1 | NM_009211.2 | fw | agcaccaaacggcatgtat | 19 | 1,94 |
|  |  | rv | ctggagcaggaggtggag | 18 |  |
| Suv39h2 | NM_022724.4 | fw | gcacgaggtgccttattca | 19 | 1,77 |
|  |  | rv | tccaatcgatttacatgtgagc | 22 |  |
| Tbpl1 | NM_011603.5 | fw | ggacccgttttatcttcatcc | 21 | 1,86 |
|  |  | rv | cccaccacgaagatcacat | 19 |  |
| Tcf7l2 | NM_001142918.1 | fw | caacgaacacagcgaatgtt | 20 | 1,99 |
|  |  | rv | ttaggagcgctcaggtctgt | 20 |  |
| Wwtr1 | NM_001168281.1 | fw | gattaggatgcgtcaagagga | 21 | 1,84 |
|  |  | rv | gtttccatggggagctgtc | 19 |  |
| Zscan21 | NM_011757.2 | fw | aaagcggttctgagtcacg | 19 | 1,94 |
|  |  | rv | gggtttctggttcttaaagtgc | 22 |  |
| Spem1 | NM_028855.1 | fw | gtattaacattggcatcaatttgg | 24 | 1,94 |
|  |  | rv | tcccaagtgaagtactggtttctt | 24 |  |
| Prm2 | NM_008933.1 | fw | gaaggcggaggagacactc | 19 | 1,84 |
|  |  | rv | ctcctccttcgggatcttct | 20 |  |
| Prm3 | NM_013638.2 | fw | agacaggagtgggcgatg | 18 | 1,87 |
|  |  | rv | gcttcttcatggaggactcg | 20 |  |
| Tnp2 | NM_013694.4 | fw | gagccttcccaccactcat | 19 | 1,95 |
|  |  | rv | tgcactggttactggtgtgact | 22 |  |
| Tekt5 | NM_001099275.1 | fw | gcagacacagctggctaaga | 20 | 1,85 |
|  |  | rv | gtattccttggccacaatgg | 20 |  |
| Adcy10 | NM_173029.3 | fw | tcttccatcatgggataaaattg | 23 | 1,83 |
|  |  | rv | gacagaaccaactccacgaac | 21 |  |
| Gapds | NM_008085.1 | fw | ccttgagatcaacacgtaccag | 22 | 1,96 |
|  |  | rv | cgcctgtacactccaccac | 19 |  |
| Catsper1 | NM_139301.2 | fw | ctgagctagagatccgaggtg | 21 | 1,95 |
|  |  | rv | caattagcttgaggactgcttct | 23 |  |
| Catsper3 | NM_029772.3 | fw | gctttctttactctcttcagtttgg | 25 | 1,88 |
|  |  | rv | cccggctcacagtaaacttc | 20 |  |
| Akap4 | NM_009651.3 | fw | ttgaggcagactggaagagtc | 21 | 1,95 |
|  |  | rv | cagtcaatatcgtcagacatcgt | 23 |  |
| Kcnu1 | NM_008432.3 | fw | gagccgacaacaactcatagc | 21 | 1,96 |
|  |  | rv | agtggtgaaatcggtcaagc | 20 |  |
| Camk4 | NM_009793.3 | fw | cgatttcttcgaggtggagt | 20 | 1,94 |
|  |  | rv | ccccttctgtttgcatctgt | 20 |  |
| Smcp | NM_008574.3 | fw | gtgctgccccaaaaagtg | 18 | 1,97 |
|  |  | rv | gtaggttgagcgcagcaag | 19 |  |
